# Supplementary material for: Extracellular vesicles engineering by silicates-activated endothelial progenitor cells for myocardial infarction treatment in male mice
Source: Nat Commun. 2023 Apr 13;14:2094. doi: 10.1038/s41467-023-37832-y (PMC10102163; doi:10.1038/s41467-023-37832-y)
Supplement: Supplementary file 1 — Supplementary Information [file 41467_2023_37832_MOESM1_ESM.pdf]

Supporting information

Extracellular vesicles engineering by silicates-activated endothelial progenitor cells for myocardial infarction treatment in male mice

Bin Yu[1,2,#], Hekai Li[3,#], Zhaowenbin Zhang[4,5,6,#], Peier Chen[3], Ling Wang[7], Xianglin Fan[3], Xiaodong Ning[1], Yuxuan Pan[1], Feiran Zhou[3], Xinyi Hu[3], Jiang Chang[1,4,5,6,**], Caiwen Ou[1,2,3,*]

[1]Guangdong Provincial Key Laboratory of Shock and Microcirculation, Dongguan Hospital of Southern Medical University (Dongguan People's Hospital), Southern Medical University, Guangzhou 510280, China

[2]Department of Rehabilitation Medicine, Zhujiang Hospital, Southern Medical University, Guangzhou, China

[3]Department of Cardiology and Laboratory of Heart Center, Zhujiang Hospital, Guangdong Provincial Biomedical Engineering Technology Research Center for Cardiovascular Disease, Southern Medical University, Guangzhou, 510515, China

[4]Wenzhou Institute, Zhejiang Engineering Research Center for Tissue Repair Materials, University of Chinese Academy of Sciences, Wenzhou, 325000, China

[5]State Key Laboratory of High-Performance Ceramics and Super fine Microstructure, Shanghai Institute of Ceramics, Chinese Academy of Sciences, Shanghai 200050, P.R. China

[6]Joint Centre of Translational Medicine, The First Affiliated Hospital of Wenzhou

Medical University, Wenzhou, 325000, China

[7]School of Biomedical Engineering, Biomaterials Research Center, Southern Medical University, Guangzhou, 510515, P.R. China

[#]These authors contributed equally: Bin Yu, Hekai Li, Zhaowenbin Zhang.

***Corresponding author:**

Email: oucaiwen@smu.edu.cn

****Co-Corresponding author:**

Email: jchang@mail.sic.ac.cn

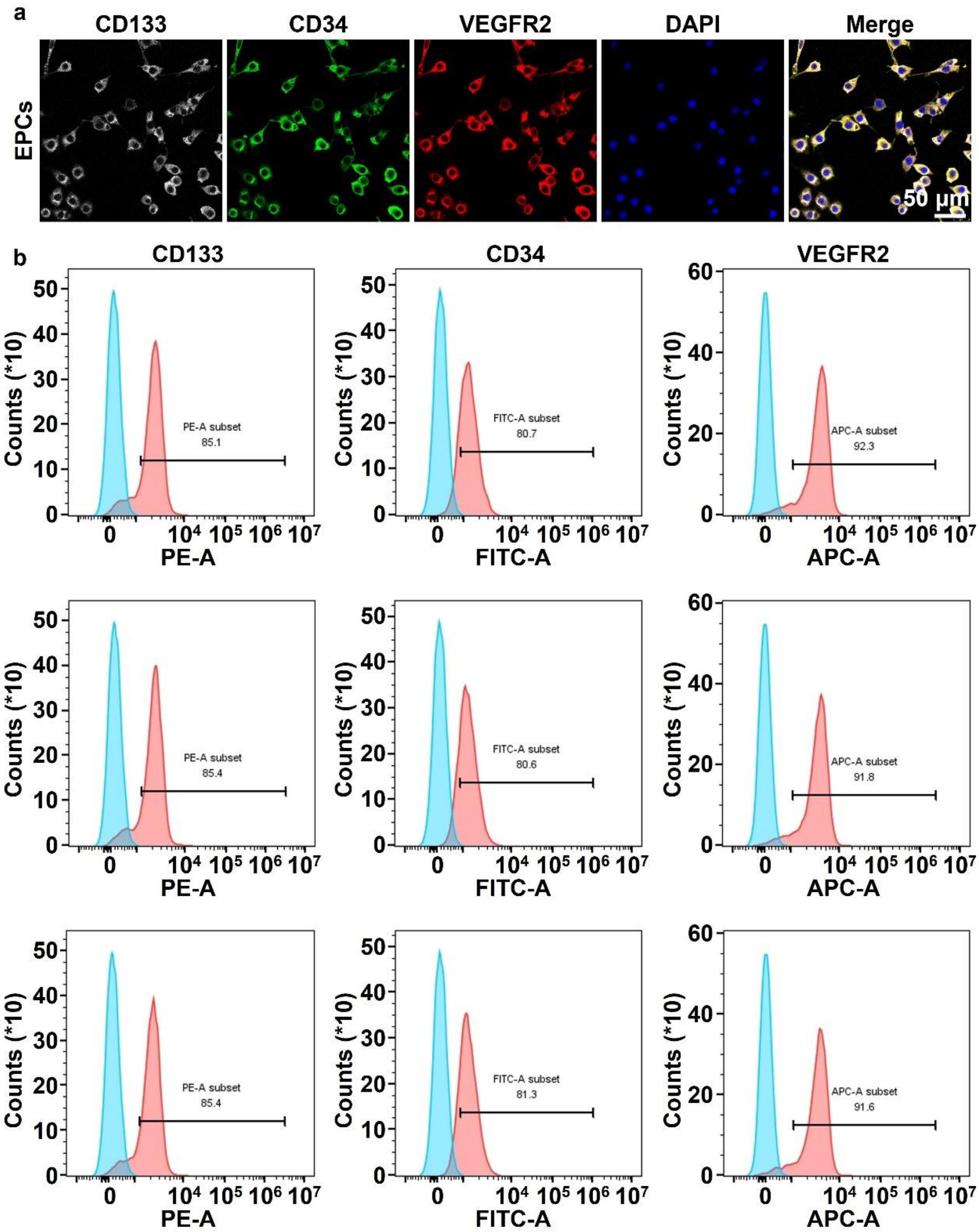

Figure S1. (a) Determination of endothelial progenitor cells (EPCs) markers by immunofluorescence staining of Cluster of Differentiation 133 (CD133), Cluster of Differentiation 34 (CD34) and Vascular Endothelial Growth Factor Receptor 2 (VEGFR2). (b) Determination of EPCs markers by flow cytometry (APC-A: VEGFR2; PE-A: CD133; FITC-A: CD34) (CD133 Positive Cells/Total Cells: 85.3±0.2%; CD34 Positive Cells/Total Cells: 80.9±0.4%; VEGFR2 Positive Cells/Total Cells: 91.9±0.4%). The 3 rows of images represent the 3 parallel results of flow cytometry analysis.

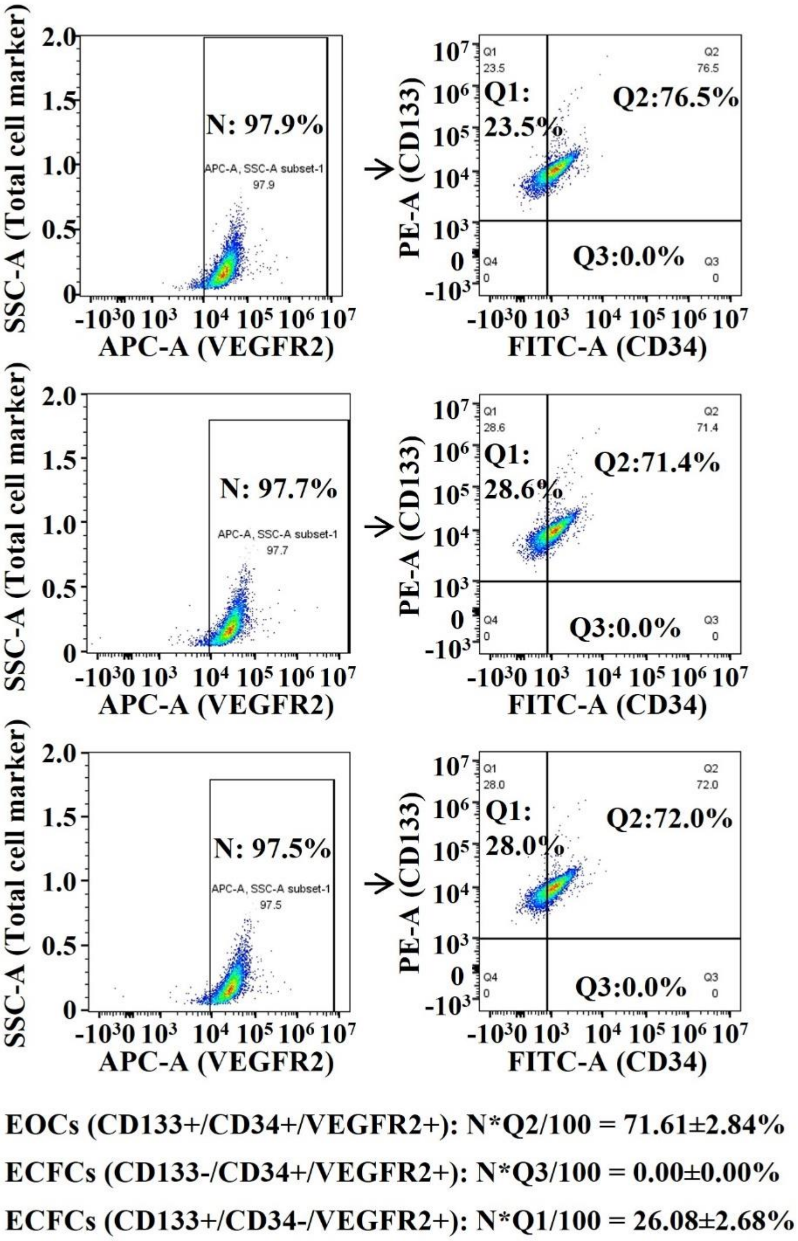

Figure S2. Flow cytometry analysis results (including FACS sequential gating strategies) of triple fluorescent labelled (APC-A: VEGFR2; PE-A: CD133; FITC-A: CD34) endothelial progenitor cells derived from mouse bone marrow. Analysis of VEGFR2 positive cells (N): CD133 positive and CD34 negative cells expression on VEGFR2 positive cells (Q1); CD133 and CD34 positive cells expression on VEGFR2 positive cells (Q2); CD34 positive and CD133 negative cells expression on VEGFR2 positive cells (Q3). The 3 rows of images represent the 3 parallel results of flow cytometry analysis.

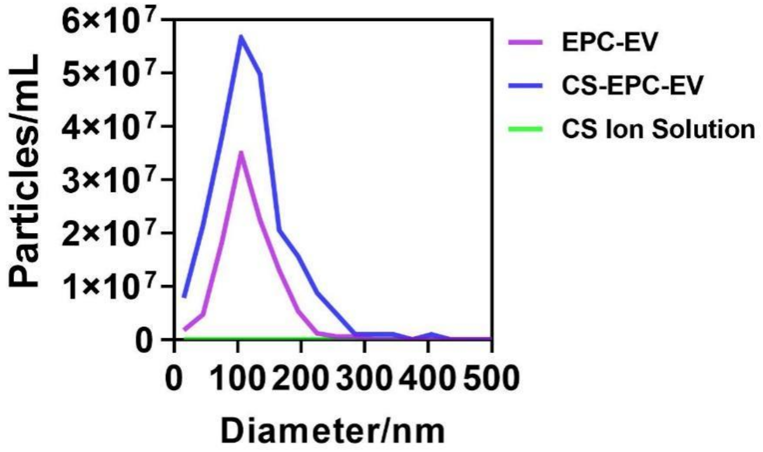

Figure S3. Nanoparticle tracking analysis (NTA) analysis of highly active extracellular vesicles (EVs) secreted from endothelial progenitor cells (EPCs) stimulated by calcium silicate (CS) ion solution (CS-EPC-EV) and normal extracellular vesicles secreted from EPCs (EPC-EV) and CS ion solution.

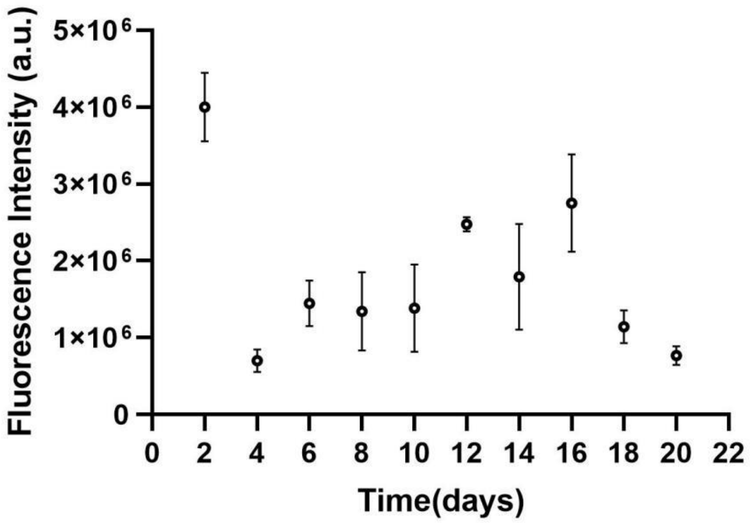

Figure S4. The fluorescence intensity of PKH26 -labeled EVs at each time when released from microspheres, n=3, independent experiment replicates per group. Data are presented as the mean ± standard.

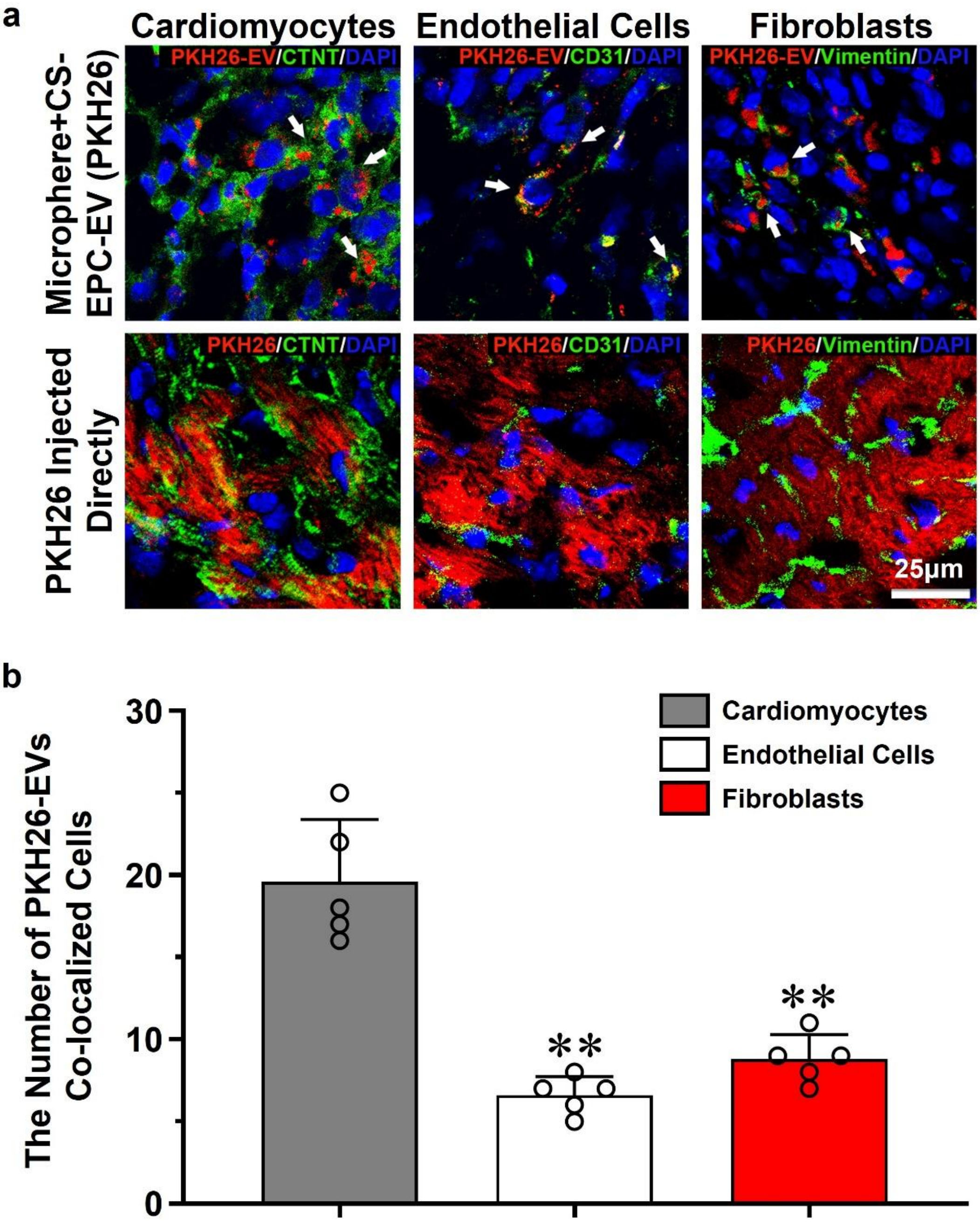

Figure S5. (a) The injected microspheres loaded with CS-EPC-EV (Microphere+CS-EPC-EV) (PKH26) were taken up by cardiomyocytes (cardiac troponin T, CTNT), endothelial cells (CD31) and fibroblasts (Vimentin). (PKH26 dye injected directly was used as control group.) (Scale Bar = 25μm). (b) The number of PKH26-EVs co-localized cells in high magnification field (60X) (Cardiomyocytes (CTAT and PKH26 co-localized): 19.6±3.8; Endothelial cells (CD31 and PKH26 co-localized): 6.6±1.1; Fibroblasts (Vimentin and PKH26 co-localized): 8.8±1.5.) (n=5, biological replicates per group). **p<0.01 vs Cardiomyocytes group. Data are presented as the mean ± standard. Two-tailed Student's t test was used to compare the differences between two

groups. One-way ANOVA and post hoc Bonferroni tests were used to compare differences among more than two groups.

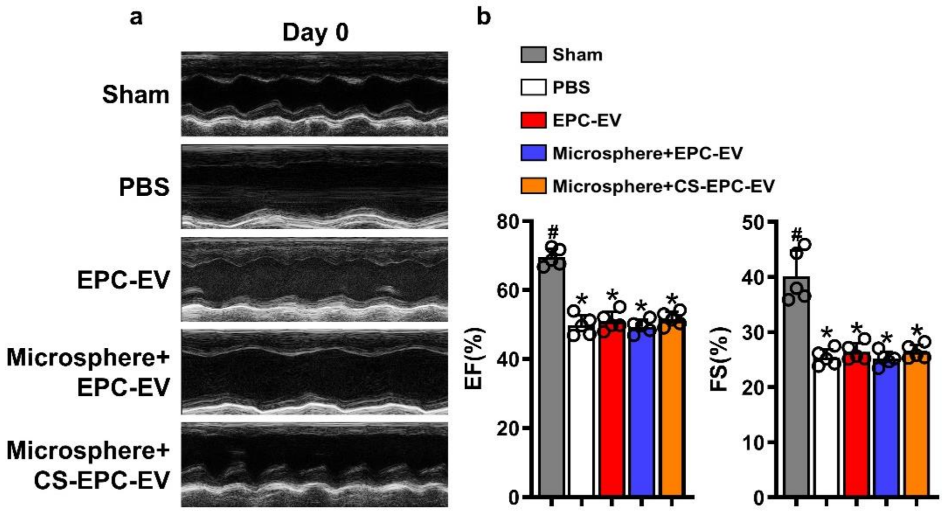

Figure S6. (a) M-mode ultrasound images on day 0 after myocardial infarction. (b) Quantification of ejection fraction (EF) and fractional shortening (FS) of the animals on day 21 after myocardial infarction. *p<0.05 vs Sham. n=5, biological replicates per group. #p<0.05 vs Microsphere+CS-EPC-EV. Data are presented as the mean ± standard. Two-tailed Student's t test was used to compare the differences between two groups. One-way ANOVA and post hoc Bonferroni tests were used to compare differences among more than two groups.

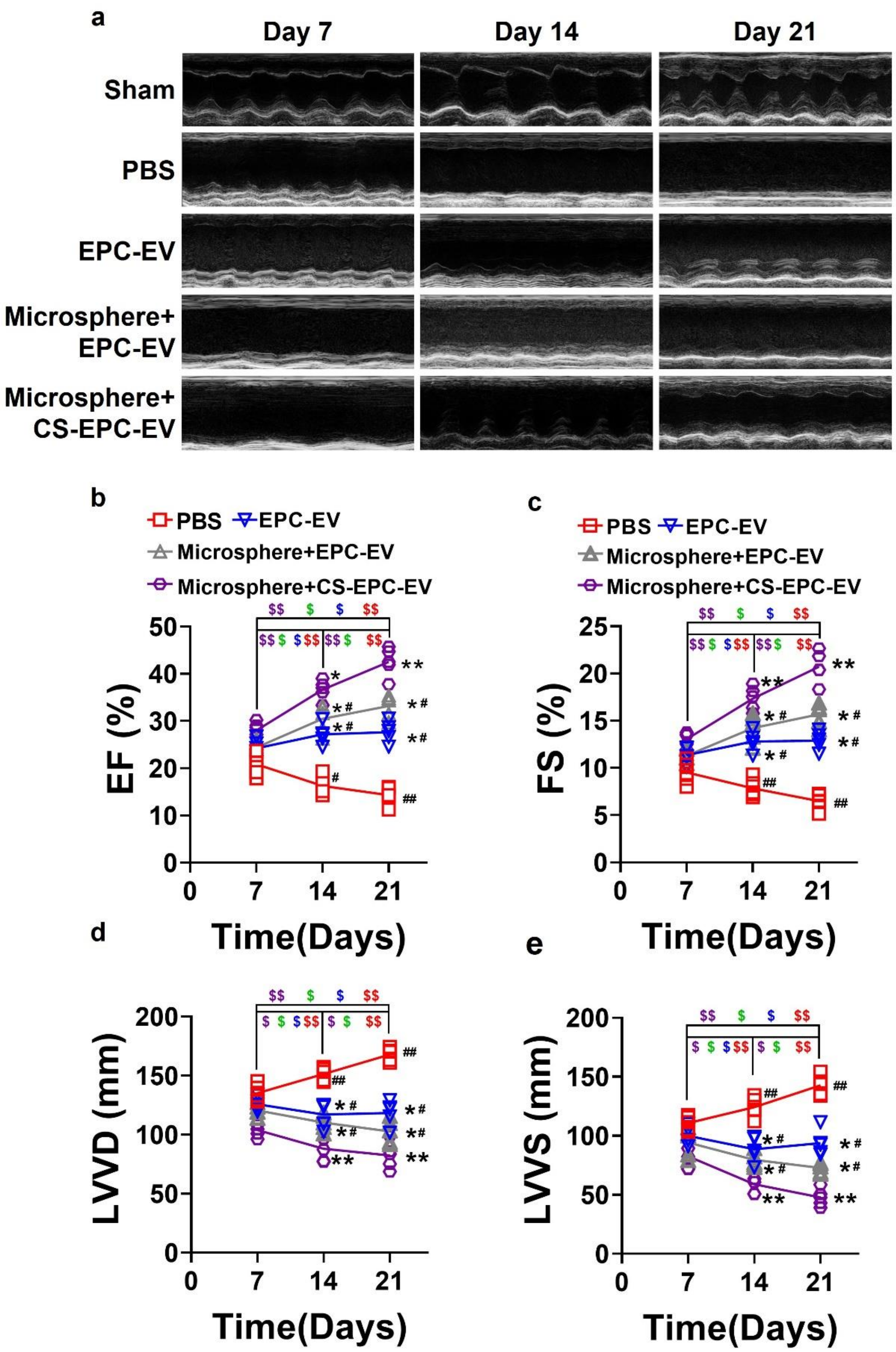

Figure S7. (a) M-mode ultrasound images on day 7, 14 and 28 after myocardial infarction. (b-e) Quantification of ejection fraction (EF), fractional shortening (FS), left ventricular end-diastolic volume (LVVD) and left ventricular end-systolic volume (LVVS) of the animals on day 7, 14 and 28 after myocardial infarction. *p<0.05, **p< 0.01 vs PBS. #p<0.05, ##p< 0.01 vs

Microsphere+CS-EPC-EV. $p<0.05, $$p< 0.01 vs different time points. n=5, biological replicates per group. Data are presented as the mean ± standard. Two-tailed Student's t test was used to compare the differences between two groups. One-way ANOVA and post hoc Bonferroni tests were used to compare differences among more than two groups. Two-way time-varying ANOVA and t tests were used to compare the time-related differences between groups.

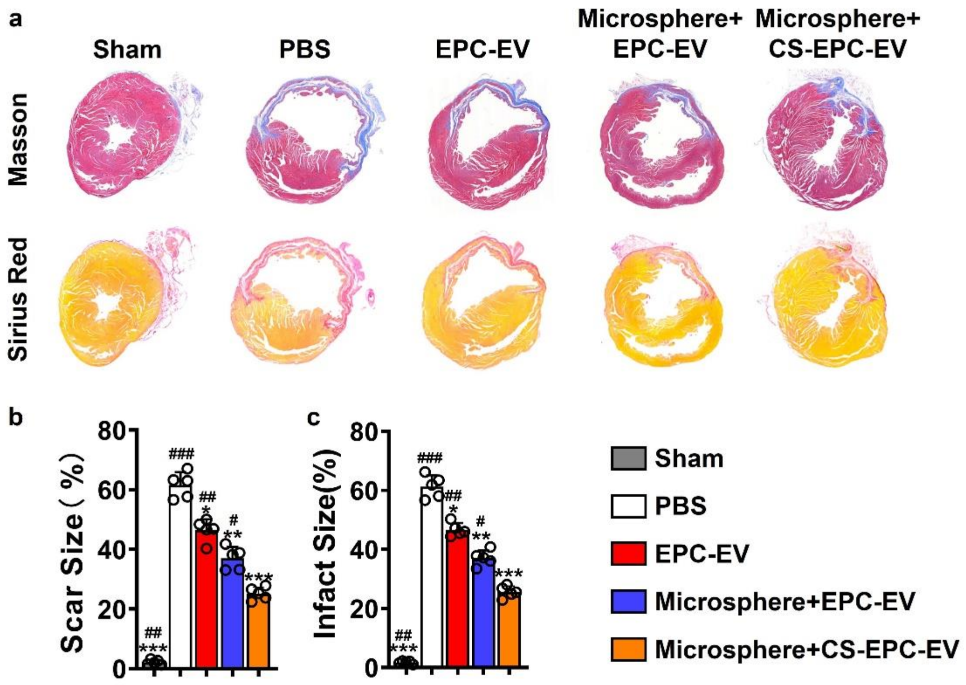

Figure S8. (a) Masson and Sirius red staining of hearts on day 21 after myocardial infarction. (b) Statistical analysis of left ventricular wall scar thickness according to the Sirius red staining. (c) Statistical analysis of left ventricular wall infarct size according to the Sirius red staining. *p<0.05, ** p<0.01 and ***p<0.001 vs PBS. #p<0.05, ##p< 0.01 and ###p<0.001 vs Microsphere+CS-EPC-EV. n=5, biological replicates per group. Data are presented as the mean ± standard. Two-tailed Student's t test was used to compare the differences between two groups. One-way ANOVA and post hoc Bonferroni tests were used to compare differences among more than two groups.

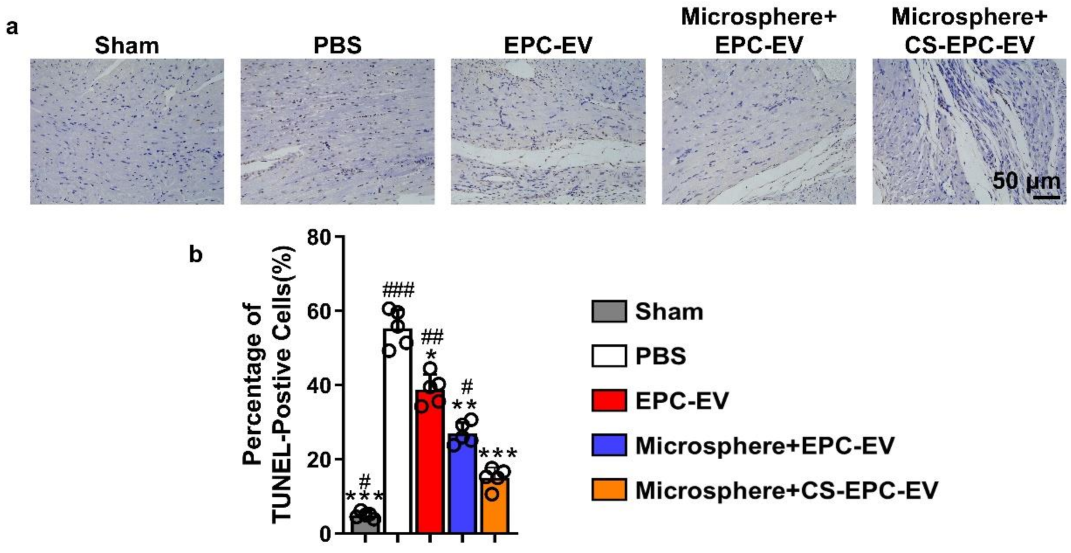

Figure S9. (a) TUNEL staining of hearts on day 21 after myocardial infarction. (b) Percentage of TUNEL-positive cells. *p<0.05, ** p<0.01 and ***p<0.001 vs PBS. #p<0.05, ##p< 0.01 and ###p<0.001 vs Microsphere+CS-EPC-EV. n=5, biological replicates per group. Data are presented as the mean ± standard. Two-tailed Student's t test was used to compare the differences between two groups. One-way ANOVA and post hoc Bonferroni tests were used to compare differences among more than two groups.

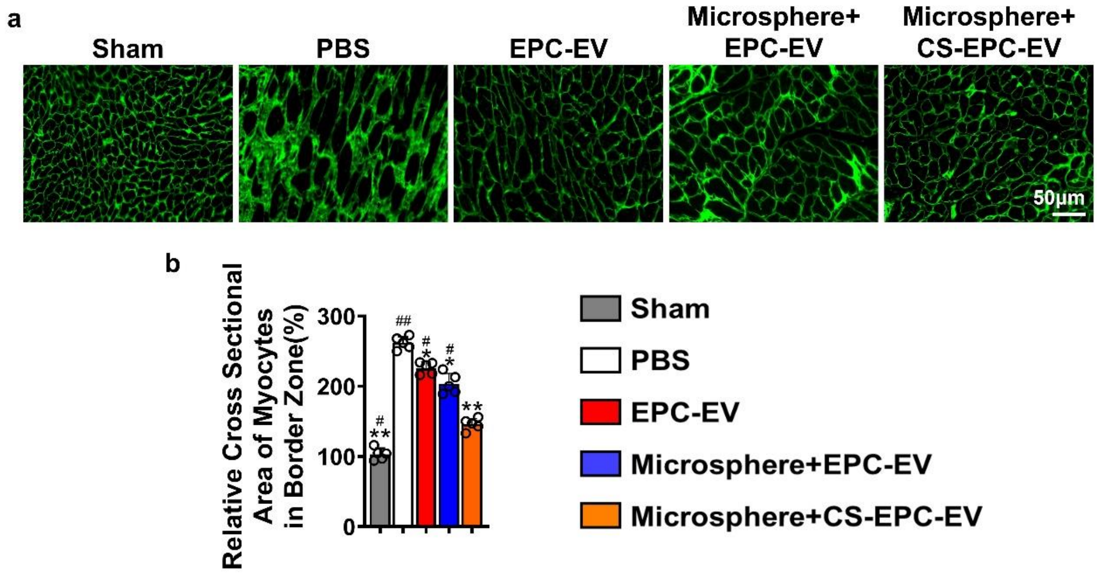

Figure S10. (a) Wheat germ agglutinin immunofluorescence staining of myocardial cells in the marginal zone of myocardial infarction on day 21 after myocardial infarction. (b) Cross-sectional

area measurements of cardiomyocytes in the marginal zone of myocardial infarction. *p<0.05, ** p<0.01 vs PBS. #p<0.05, ##p< 0.01 vs Microsphere+CS-EPC-EV. n=5, biological replicates per group. Data are presented as the mean ± standard. Two-tailed Student's t test was used to compare the differences between two groups. One-way ANOVA and post hoc Bonferroni tests were used to compare differences among more than two groups.

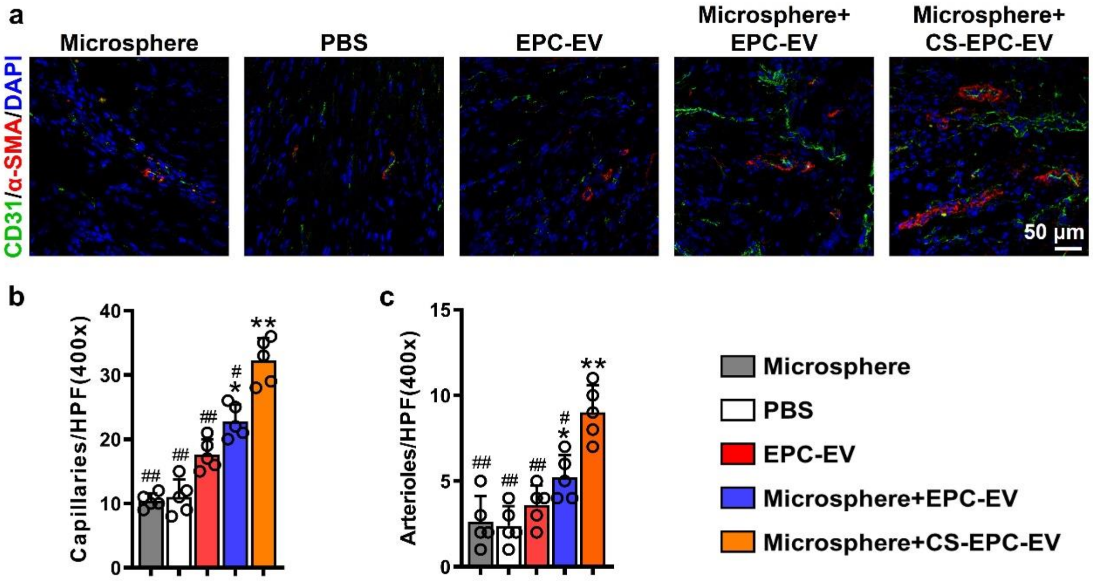

Figure S11. (a) CD31 (green fluorescence) and α-SMA (red fluorescence) staining of hearts on day 21 after myocardial infarction. (b) Statistical analysis of capillaries. (c) Statistical analysis of arterioles. *p<0.05, ** p<0.01 vs PBS. #p<0.05, ##p< 0.01 vs Microsphere+CS-EPC-EV. n=5, biological replicates per group. Data are presented as the mean ± standard. Two-tailed Student's t test was used to compare the differences between two groups. One-way ANOVA and post hoc Bonferroni tests were used to compare differences among more than two groups.

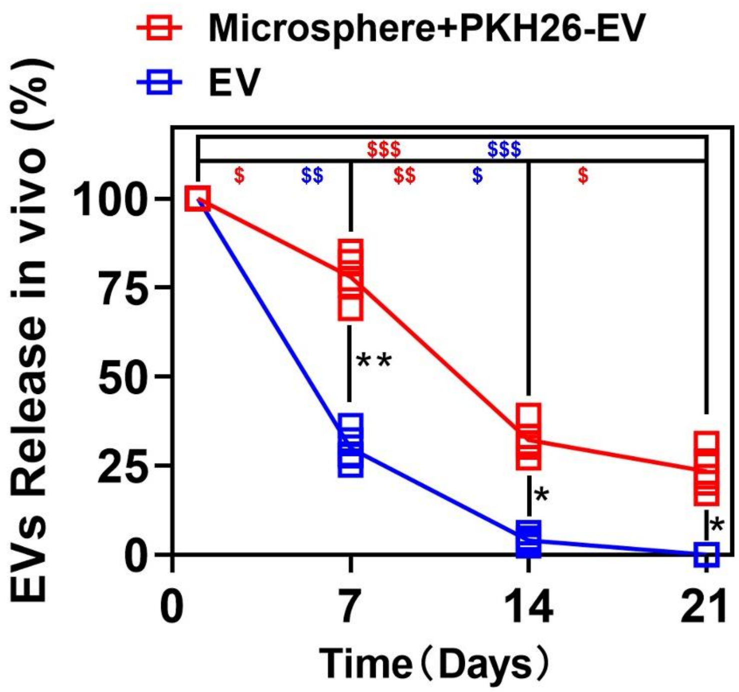

Figure S12. According to the fluorescence intensity of the PKH26-EV *in vivo* (Figure 5a), quantitative analysis of the EVs release from microspheres for 21 days (n=5, biological replicates per group). *p<0.05, ** p<0.01 vs EV. $p<0.05, $$p< 0.01, $$$p< 0.001 vs different time points. Data are presented as the mean ± standard. Two-tailed Student's t test was used to compare the differences between two groups. One-way ANOVA and post hoc Bonferroni tests were used to compare differences among more than two groups. Two-way time-varying ANOVA and t tests were used to compare the time-related differences between groups.

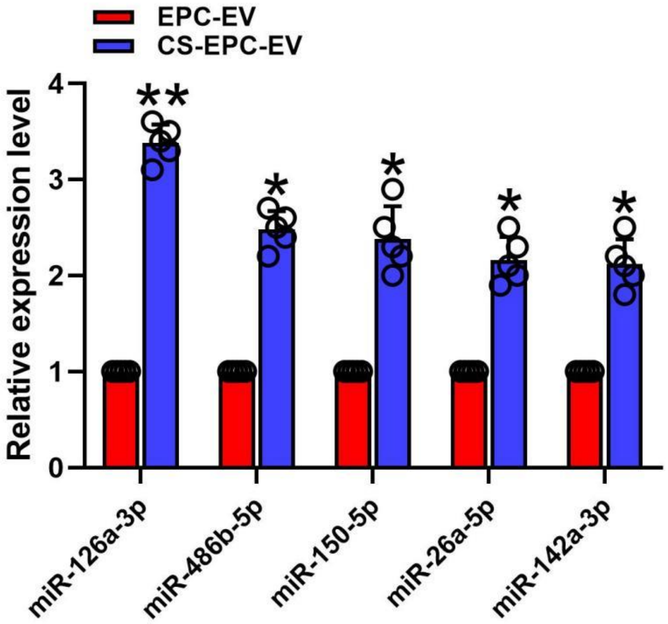

Figure S13. RT-qPCR analysis of the miRNA expression level of miR-126a-3p, miR-486b-5p, miR-150-5p, miR-26a-5p, miR-142a-3p in EPC-EV or CS-EPC-EV. *p< 0.05, **p< 0.01 vs EPC-EV, n=5, biological replicates per group. Data are presented as the mean ± standard. Two-tailed Student's t test was used to compare the differences between two groups. One-way ANOVA and post hoc Bonferroni tests were used to compare differences among more than two groups.

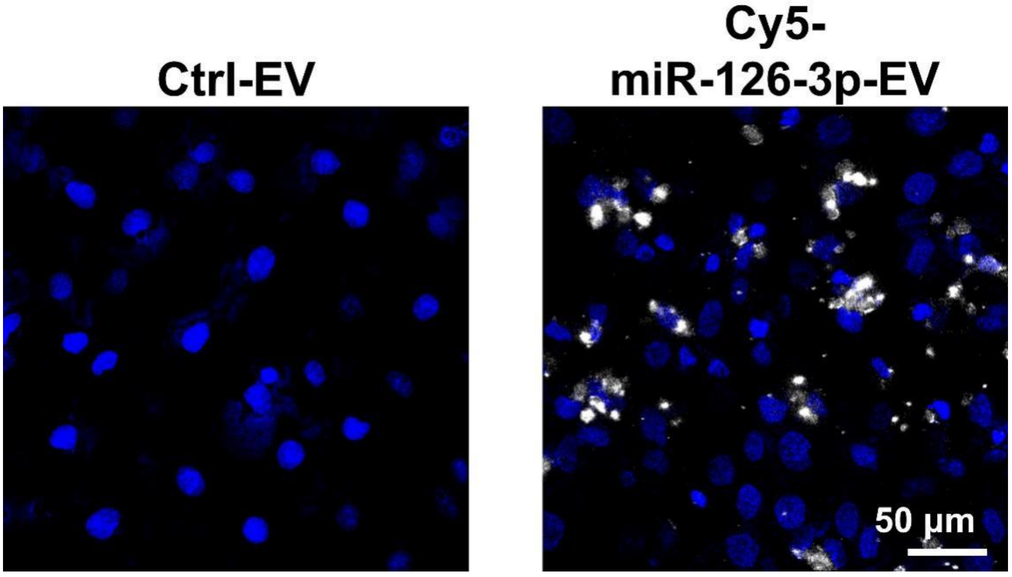

Figure S14. The miR-126a-3p could transfer from EPCs to HUVECs through EVs.

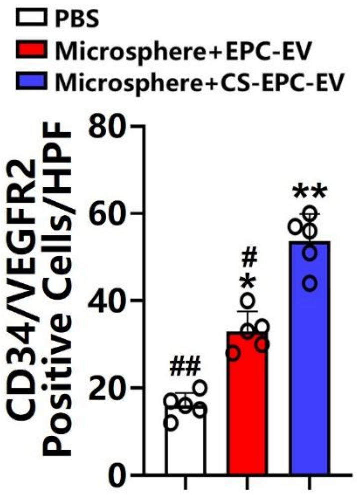

Figure S15. Quantitative analysis of Figure 6c of CD34+/VEFGFR2+ positive cells., *p<0.05, **p< 0.01 vs PBS group. #p<0.05, ##p< 0.01 vs Microsphere+CS-EPC-EV. n=5, biological replicates per group. Data are presented as the mean ± standard. Two-tailed Student's t test was used to compare the differences between two groups. One-way ANOVA and post hoc Bonferroni tests were used to compare differences among more than two groups.

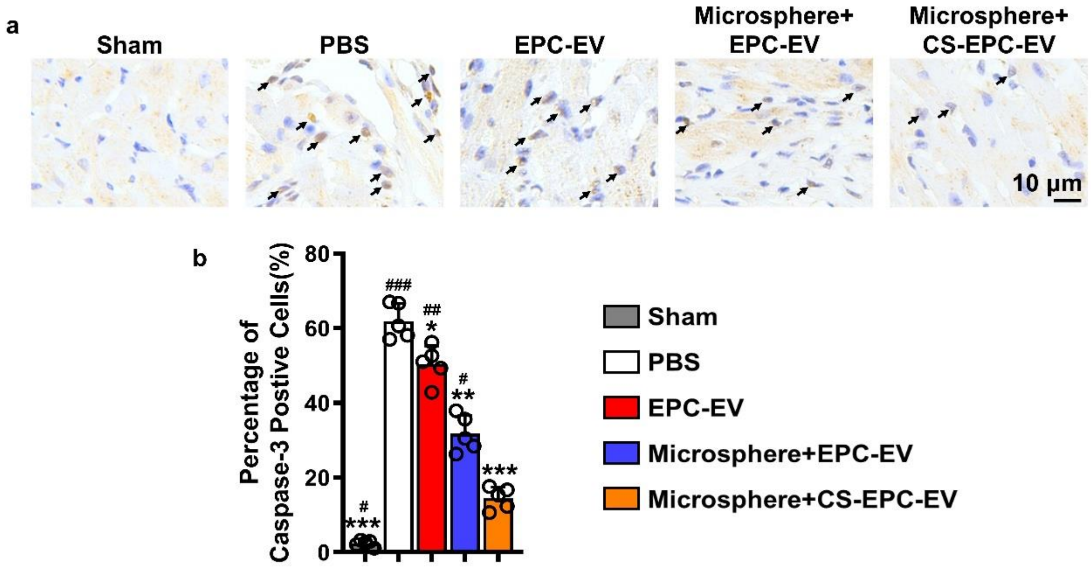

Figure S16. (a) Caspase 3 staining of hearts on day 21 after myocardial infarction. (b) Percentage of Caspase-positive cells. *p<0.05, ** p<0.01 and ***p<0.001 vs PBS. #p<0.05, ##p<0.01 and ###p<0.001 vs Microsphere+CS-EPC-EV. n=5, biological replicates per group. Data are presented as the mean ± standard. Two-tailed Student's t test was used to compare the differences between two groups. One-way ANOVA and post hoc Bonferroni tests were used to compare differences among more than two groups.

Table S1. The ion concentration in calcium silicate (CS) extracts. *$p < 0.05$ VS the data of the same ions in control. n = 3. Data are presented as the mean ± standard. Two-tailed Student's t test was used to compare the differences between two groups. One-way ANOVA and post hoc Bonferroni tests were used to compare differences among more than two groups.

	Si (μg/mL)	Ca (μg/mL)	P (μg/mL)
Control	0.33±0.03	60.87±0.12	17.31±0.29
1/64CS	1.84±0.15*	60.22±0.50	16.79±0.14
1/128CS	1.26±0.11*	60.56±0.48	16.62±0.24
1/256CS	0.67±0.08*	61.04±0.49	16.56±0.25

Table S2. Primer sequences for RT-qPCR.

Mouse:

**Primers**	Forward (5'-3')	Reverse (5'-3')
VEGFA	CTCGTGGGACTGGATTCGC	CCAACACAAGTCCACAGCAGTC
eNOS	CGCAAGAGGAAGGAGTCTAGCA	TCGAGCAAAGGCACAGAAGTGG
SDF-1	GGAGGATAGATGTGCTCTGGAAC	AGTGAGGATGGAGACCGTGGTG
IGF-1	GGCTGGCTAGCAAAGGTGTGG	GACTTAATGATCTTTGTGGGGAA TGGG
SMPD3	TGACTGGAAGGCTGAGGTAGA	ATTGATGGGCTCGTCCTTCC
Syncrip	GGCAAGACGTAGGCTAATGAGTG	TACCGTGTTGGCAAGGTTGCGT
Ybx1	CAGGAGAGCAAGGTAGACCAGT	TGCTGACCTTGGGTCTCATCTC
ANXA2	CACCAACTTCGATGCTGAGAGG	GCACATTGCTGCGGTTTGTCAG
hnRNPA2B1	CGGTGGCAATTTTGGACCAGGA	CCATAACCAGGGCTACCTCCAA
GAPDH	CATCACTGCCACCCAGAAGACTG	ATGCCAGTGAGCTTCCCGTTCAG

Human:

VEGFA	TTCCTGTAGACACACCCACC	CAGGGATTTTTCTTGTCTTGCT
bFGF	CAATTCCCATGTGCTGTGAC	ACCTTGACCTCTCAGCCTCA

eNOS	GAAGGCGACAATCCTGTATGGC	TGTTCGAGGGACACCACGTCAT
SDF-1	TCAACACTCCAAACTGTGCCC	CTCCAGGTACTCCTGAATCCAC
IGF-1	CACCAACTTCGATGCTGAGAGG	GCACATTGCTGCGGTTTGTCAG
GAPDH	CTCTTCAGTTCGTGTGTGGAGAC	CAGCCTCCTTAGATCACAGCTC

Table S3. The mass of different angiogenic factor proteins in 1μg extracellular vesicles.

The Mass of Different Angiogenic Factor Proteins in 1μg Extracellular vesicles (μg)			
Angiogenic Factor Proteins	Number	EPC-EV	CS-EPC-EV
VEGFA	1	$2.47*10^{-6}$	$6.12*10^{-6}$
	2	$2.14*10^{-6}$	$6.92*10^{-6}$
	3	$2.56*10^{-6}$	$4.78*10^{-6}$
	Mean ± Standard	$(2.39±0.22)*10^{-6}$	$(5.94±1.08)*10^{-6}$
SDF-1	1	$7.70*10^{-5}$	$1.62*10^{-4}$
	2	$9.43*10^{-5}$	$1.75*10^{-4}$
	3	$8.86*10^{-5}$	$1.59*10^{-4}$
	Mean ± Standard	$(8.66±0.88)*10^{-5}$	$(1.65±0.08)*10^{-4}$
IGF-1	1	$3.78*10^{-7}$	$5.33*10^{-7}$
	2	$3.90*10^{-7}$	$5.64*10^{-7}$
	3	$3.54*10^{-7}$	$5.24*10^{-7}$
	Mean ± Standard	$(3.74±0.18)*10^{-7}$	$(5.40±0.21)*10^{-7}$
eNOS	1	$2.64*10^{-4}$	$4.05*10^{-4}$
	2	$2.58*10^{-4}$	$3.42*10^{-4}$
	3	$2.32*10^{-4}$	$4.64*10^{-4}$
	Mean ± Standard	$(2.51±0.17)*10^{-4}$	$(4.03±0.61)*10^{-4}$
HGF	1	$1.66*10^{-2}$	$3.00*10^{-2}$
	2	$1.53*10^{-2}$	$2.82*10^{-2}$
	3	$1.79*10^{-2}$	$2.95*10^{-2}$
	Mean ± Standard	$(1.66±0.13)*10^{-2}$	$(2.91±0.09)*10^{-2}$

Table S4. The number of particles in extracellular vesicles.

The Number of Particles in 1μg EVs

	EPC-EV	CS-EPC-EV
1	$2.32*10^9$	$2.44*10^9$
2	$2.47*10^9$	$2.26*10^9$
3	$2.11*10^9$	$2.39*10^9$
Mean ± Standard	$(2.30±0.18)*10^9$	$(2.36±0.09)*10^9$
The Number of Particles Injected into the Myocardial Infarction Site		
	EPC-EV	CS-EPC-EV
Mean ± Standard	$(46.0±3.6)*10^9$	$(47.3±1.8)*10^9$

Table S5. The angiogenic factor content (μg) in one extracellular vesicle.

The Angiogenic Factor Content (μg) in one Extracellular vesicle		
Angiogenic Factor Proteins	EPC-EV	CS-EPC-EV
VEGFA	$(1.05±0.17)*10^{-15}$	$(2.52±0.53)*10^{-15}$
SDF-1	$(3.78±0.44)*10^{-14}$	$(0.70±0.06)*10^{-13}$
IGF-1	$(1.63±0.05)*10^{-16}$	$(2.29±0.18)*10^{-16}$
eNOS	$(1.09±0.05)*10^{-13}$	$(1.70±0.22)*10^{-13}$
HGF	$(0.73±0.11)*10^{-11}$	$(1.24±0.01)*10^{-11}$

Table S6. The content of different angiogenic factor proteins in extracellular vesicles injected in each mouse (20μg extracellular vesicles).

The Mass of Different Angiogenic Factor Proteins Injected in Each Mouse (20μg Extracellular vesicles)		
Angiogenic Factor Proteins	EPC-EV	CS-EPC-EV
VEGFA	$(47.80±4.42)*10^{-6}$	$(118.80±21.63)*10^{-6}$
SDF-1	$(17.33±1.76)*10^{-4}$	$(33.07±1.70)*10^{-4}$
IGF-1	$(74.80±3.67)*10^{-7}$	$(108.07±4.20)*10^{-7}$
eNOS	$(50.27±3.40)*10^{-4}$	$(80.73±12.20)*10^{-4}$
HGF	$(33.20±2.60)*10^{-2}$	$(58.47±1.86)*10^{-2}$

Table S7. The purity of EVs

EPC-EV

	The Number of Particles (Particles/mL)	Protein Content (μg/mL)	Purity Ratio (Particles/μg)
1	$1.30*10^9$	0.6	$2.17*10^9$
2	$1.10*10^9$	0.47	$2.34*10^9$
3	$1.00*10^9$	0.41	$2.44*10^9$
Mean ± Standard	$(1.13±0.15)*10^9$	0.49±0.10	$(2.32±0.14)*10^9$
CS-EPC-EV			
	The Number of Particles (Particles/mL)	Protein Content (μg/mL)	Purity Ratio (Particles/μg)
1	$1.90*10^9$	0.8	$2.38*10^9$
2	$2.30*10^9$	0.87	$2.64*10^9$
3	$1.70*10^9$	0.75	$2.27*10^9$
Mean ± Standard	$(1.97±0.31)*10^9$	0.81±0.06	$(2.43±0.19)*10^9$
