# Supplementary material for: Extracellular vesicles engineering by silicates-activated endothelial progenitor cells for myocardial infarction treatment in male mice
Source: Nat Commun. 2023 Apr 13;14:2094. doi: 10.1038/s41467-023-37832-y (PMC10102163; doi:10.1038/s41467-023-37832-y)
Supplement: Supplementary file 3 — Description of Additional Supplementary Files [file 41467_2023_37832_MOESM3_ESM.pdf]

Description of Additional Supplementary Files

Supplementary Movie 1: Fluorescence scanning through one Microsphere+EVs from top to bottom using a confocal laser scanning microscope, which shows the uniform distribution of extracellular vesicles within the microsphere. Microspheres (FITC-labeled, green fluorescence), EVs (PKH26-labeled, red fluorescence).
