# Supplementary material for: Extracellular vesicles engineering by silicates-activated endothelial progenitor cells for myocardial infarction treatment in male mice
Source: Nat Commun. 2023 Apr 13;14:2094. doi: 10.1038/s41467-023-37832-y (PMC10102163; doi:10.1038/s41467-023-37832-y)
Supplement: Supplementary file 5 — Reporting Summary [file 41467_2023_37832_MOESM5_ESM.pdf]

nature portfolio

Corresponding author(s): Jiang Chang; Caiwen Ou

Last updated by author(s): Mar 19, 2023

Reporting Summary

Nature Portfolio wishes to improve the reproducibility of the work that we publish. This form provides structure for consistency and transparency in reporting. For further information on Nature Portfolio policies, see our Editorial Policies and the Editorial Policy Checklist.

Statistics

For all statistical analyses, confirm that the following items are present in the figure legend, table legend, main text, or Methods section.

n/a	Confirmed	
☐	☒	The exact sample size (*n*) for each experimental group/condition, given as a discrete number and unit of measurement
☐	☒	A statement on whether measurements were taken from distinct samples or whether the same sample was measured repeatedly
☐	☒	The statistical test(s) used AND whether they are one- or two-sided<br>*Only common tests should be described solely by name; describe more complex techniques in the Methods section.*
☐	☒	A description of all covariates tested
☐	☒	A description of any assumptions or corrections, such as tests of normality and adjustment for multiple comparisons
☐	☒	A full description of the statistical parameters including central tendency (e.g. means) or other basic estimates (e.g. regression coefficient) AND variation (e.g. standard deviation) or associated estimates of uncertainty (e.g. confidence intervals)
☐	☒	For null hypothesis testing, the test statistic (e.g. *F*, *t*, *r*) with confidence intervals, effect sizes, degrees of freedom and *P* value noted<br>*Give P values as exact values whenever suitable.*
☒	☐	For Bayesian analysis, information on the choice of priors and Markov chain Monte Carlo settings
☒	☐	For hierarchical and complex designs, identification of the appropriate level for tests and full reporting of outcomes
☒	☐	Estimates of effect sizes (e.g. Cohen's *d*, Pearson's *r*), indicating how they were calculated

Our web collection on statistics for biologists contains articles on many of the points above.

Software and code

Policy information about availability of computer code

Data collection	ImageJ (Version 1.5.3), FlowJo (Version 10.4) were used to collect data in this study.
Data analysis	Fluorescence and bioluminescece images were analyzed using the LeicaSP8(Advanced Fluorescence 4.0.0.11706). Pathological images were acquired with EasyScan 6(Motic EasyScanner). Statistical calculationswere pertormed using GraphPad Prism 8 software,The Western blot were performed using ImageQuant LAS500(ImageQuant™ TL). The data anlaysis were performed using ImageJ. Flow cytometr analysis were nerformed using CytoidFLEX instrument by CytExpert software, and analysed using FlowJo-v1 0.8.1.

For manuscripts utilizing custom algorithms or software that are central to the research but not yet described in published literature, software must be made available to editors and reviewers. We strongly encourage code deposition in a community repository (e.g. GitHub). See the Nature Portfolio guidelines for submitting code & software for further information.

Data

Policy information about availability of data

All manuscripts must include a data availability statement. This statement should provide the following information, where applicable:

- Accession codes, unique identifiers, or web links for publicly available datasets
- A description of any restrictions on data availability
- For clinical datasets or third party data, please ensure that the statement adheres to our policy

All data needed to evaluate the conclusions in the paper are present in the paper and/or the Supplementary Information. Source data are provided with this paper. Additional data have been uploaded to the science data bank (https://www.scidb.cn/s/Rjamqm) in its original form. The data includes statistical graphs related to

cell viability, cell migration, cell ring formation, quantitative analysis of animal experiments, Western Blot bands, and Western Blot parallel samples. The sequencing results have been deposited to the NCBI SRA public database under the accession number: PRJNA944988, and hyperlink: https://www.ncbi.nlm.nih.gov/bioproject/PRJNA944988, and CS-EPC-EV SRA accession number: SRX19681860; SRX19681859; SRX19681858, and EPC-EV SRA accession number: SRX19681857; SRX19681856; SRX19681855.

Human research participants

Policy information about studies involving human research participants and Sex and Gender in Research.

Reporting on sex and gender	No human research participants in this study.
Population characteristics	See above.
Recruitment	See above.
Ethics oversight	See above.

Note that full information on the approval of the study protocol must also be provided in the manuscript.

Field-specific reporting

Please select the one below that is the best fit for your research. If you are not sure, read the appropriate sections before making your selection.

☒ Life sciences ☐ Behavioural & social sciences ☐ Ecological, evolutionary & environmental sciences

For a reference copy of the document with all sections, see nature.com/documents/nr-reporting-summary-flat.pdf

Life sciences study design

All studies must disclose on these points even when the disclosure is negative.

Sample size	No sample size calculations were performed. The sample sizes were determined based on previous literature, allowing for statistical analyses such as calculation of standard deviation and performing t-tests. In vitro studies were repeated a minimum of two to three times for independence and the in vivo sample sizes were determined following established standards for animal studies, with a minimum of n=3 biological replicates for adequate reproducibility.
Data exclusions	No data were excluded from the analyses.
Replication	All cell experiments were repeated 3 times, and all animal experiments were repeated 2 times. All experiments were reproduced to reliably support conclusions stated in the manuscript.
Randomization	Samples/organisms/paryicipants were allocated into experimental groups randomly.
Blinding	All the results were assessed blindly by three people in this study.

Reporting for specific materials, systems and methods

We require information from authors about some types of materials, experimental systems and methods used in many studies. Here, indicate whether each material, system or method listed is relevant to your study. If you are not sure if a list item applies to your research, read the appropriate section before selecting a response.

Materials & experimental systems

n/a	Involved in the study	
☐	☒	Antibodies
☐	☒	Eukaryotic cell lines
☒	☐	Palaeontology and archaeology
☐	☒	Animals and other organisms
☒	☐	Clinical data
☒	☐	Dual use research of concern

Methods

n/a	Involved in the study	
☒	☐	ChIP-seq
☐	☒	Flow cytometry
☒	☐	MRI-based neuroimaging

Antibodies

Antibodies used	Staining: CD31 (1:500, Servicebio, GB13063), α-SMA (1:500, Abcam, ab124964), CD34 (1:500, Abcam, ab8158) and VEGFR2 (1:500, Abcam, ab214424), CD68 (1:500, CST, 29176), CTNT (1:500, Abcam, ab209813), Vimentin (1:500, Abcam, ab92547), caspase 3 (1:500, Abcam, ab184787).Donkey Anti-Goat IgG H&L (Abcam, ab6949),Donkey Anti-Rabbit IgG H&L (Abcam, ab150073),Donkey Anti-Goat IgG H&L (Abcam, ab150135),Donkey Anti-Rat IgG H&L (Abcam, ab150153),Donkey Anti-Rabbit IgG H&L (Abcam,ab150075) WesternBlot/ELISA:ALIX(Abcam,ab275377),CD81(Abcam,ab109201),CD63(Abcam, ab217345) ,eNOS (1:5000, Abcam, ab300071), VEGFA (1:2000, Abcam, ab214424), SDF-1 (1:2000, Abcam, ab25117), AKT (1:2000, CST, 9272), p-AKT (1:2000, CST,4060), β-actin (1:1000, Abcam, ab8226), RGS16 (1:2000, Abcam, ab119424), CXCR4 (1:2000, Abcam, ab181020), GAPDH(1:5000, Abcam, ab8245), hnRNPA2B1(1:2000, Abcam, ab183654),NSMase2(1:2000, Abcam, ab85017),Goat Anti-Rabbit IgG H&L(Abcam, ab205718),Flow Cytometry: CD133 (Biolegend, 141203), CD34 (BD, 560238), VEGFR2 (Thermo, 17-5821-81).
Validation	All antibodies were validated by testing the secondary antibodies in isolation. For immunohistochemistry, the appropriate cellular localization of the signal (membrane-bound, nuclear, cytoplasmic) was confirmed, and for western blotting, the correct size of the signal further validated the antibodies. Validation information for each of the antibodies used in the study can be found on the manufacturer's website.

Eukaryotic cell lines

Policy information about cell lines and Sex and Gender in Research

Cell line source(s)	Human umbilical vein endothelial cells were purchased from iCellbioscience.
Authentication	All cell lines were authenticated by the supplier using Short Tandem Repeat test.
Mycoplasma contamination	All cell lines tested were negative for mycoplasma contamination.
Commonly misidentified lines (See ICLAC register)	No commonly misidentified cell lines were used.

Animals and other research organisms

Policy information about studies involving animals; ARRIVE guidelines recommended for reporting animal research, and Sex and Gender in Research

Laboratory animals	C57BL/6 mice and BALB/c nude mice of 8 weeks of age (average weight is 20g) were purchased from the Laboratory Animal Center of Southern Medical University. The mice were grouped and housed in a controlled environment with a 12-hour light-dark cycle, ambient temperature ranging from 18 to 22°C, and 50-70% humidity. They were provided with unrestricted access to food and water.
Wild animals	The study did not involve wild animals.
Reporting on sex	Findings apply to only male mice.
Field-collected samples	Samples were collected under room temperature.
Ethics oversight	This experimental protocol was approved by the Animal Care and Use Committee of Southern Medical University (LAEC-2021-076) and complied with the Guide for the Care and Use of Laboratory Animals published by the National Institutes of Health (8th edition, 2011).

Note that full information on the approval of the study protocol must also be provided in the manuscript.

Flow Cytometry

Plots

Confirm that:

- [x] The axis labels state the marker and fluorochrome used (e.g. CD4-FITC).
- [x] The axis scales are clearly visible. Include numbers along axes only for bottom left plot of group (a 'group' is an analysis of identical markers).
- [x] All plots are contour plots with outliers or pseudocolor plots.
- [x] A numerical value for number of cells or percentage (with statistics) is provided.

Methodology

Sample preparation	First, mouse bone marrow derived endothelial progenitor cells (EPC) were cultured for 24 hours. The trypsin was then used to digest the cells and centrifuged. PBS was used for cell suspension preparation. The cells were divided into the following groups: blank tube, CD34 positive tube, CD133 positive tube, and CD309 positive tube. According to the grouping, the corresponding antibody (1:50) was added and incubated in dark for 30 min. After the reaction was terminated, the computer

	was used for detection.
Instrument	Cytoflex S flow cytometry, manufactured by Beckman.
Software	CytExpert software collects cell data and uses FlowJo10.4 to analyze the data.
Cell population abundance	According to the flow analysis results, 80.9% of the cells were CD34 positive, 85.3% were CD133 positive, and 91.9% were CD309 positive. They highly expressed endothelial progenitor cell markers, so it proved that the vast majority of our cell population was endothelial progenitor cells.
Gating strategy	According to FSC-A and SSC-A, we select the total cell population, and then select the single cell according to FSC-A and FSC-H. Then, we circle the positive gate according to the negative principle of blank tube, and then analyze the positive proportion of CD34, CD133 and CD309 in CD34 positive tube, CD133 positive tube and CD309 positive tube respectively under the condition of unchanged gating, and finally convert them into a single parameter histogram.

☒ Tick this box to confirm that a figure exemplifying the gating strategy is provided in the Supplementary Information.
